# Impact of the COVID-19 pandemic on the consumption of antibiotics and the emergence of AMR: case study in a general hospital

**DOI:** 10.3389/fpubh.2025.1584574

**Published:** 2025-06-23

**Authors:** Urška Rozman, Konrad Kranjec, Aleksander Šeruga, Urška Kramar, Dominika Vrbnjak, Miha Lavrič, Sonja Šostar Turk

**Affiliations:** ^1^Faculty of Health Sciences, University of Maribor, Maribor, Slovenia; ^2^General Hospital Ptuj, Ptuj, Slovenia; ^3^National Laboratory of Health, Environment and Food, Maribor, Slovenia

**Keywords:** COVID-19, antibiotics, AMR, general hospital, Slovenia

## Abstract

**Objectives:**

The COVID-19 pandemic changed the use of antibiotics and had an impact on the development of antimicrobial resistance. The study aimed to examine the consumption of antibiotics and the occurrence of AMR infection and colonization in the selected general hospital.

**Methods:**

Data on antibiotic consumption and data on AMR infections and colonization were monitored in the period before the COVID-19 pandemic (2018, 2019) and during the COVID-19 pandemic (2020, 2021). Descriptive statistics, the Mann–Whitney U test, and the Pearson or Spearman correlation test were used.

**Results:**

The overall prescription of antibiotics stayed approximately the same, however, some important differences can be observed when analyzing specific groups of antibiotics (vancomycin, linezolid, piperacillin/tazobactam, meropenem, colistin). We did not observe the difference in the occurrence of AMR infections and colonizations before and during the pandemic. However, we did observe an alarming increase in CRaB, ESBL and VRE and highlighted the increase in all AMR groups between the first and second year of the pandemic.

**Conclusion:**

The connection between antibiotic consumption and the occurrence of AMR infections and colonization was confirmed.

## Introduction

On the 5th of May, 2023, after more than 3 years, the World Health Organization (WHO) declared Coronavirus disease 2019 (COVID-19) no longer a global health emergency and classified it as an established and ongoing health issue (1). Nevertheless, many studies emphasize the importance of changed or increased use of antibiotics during the pandemic and the impact on the development of antimicrobial resistance (2–8). The COVID-19 pandemic has required changes in disease prevention protocols, including the wearing of protective equipment and masks, hand washing and sanitizing, social distancing (9) and also changes in hospital guidelines, introduction of COVID-19 screening tests and patient isolation (10). On October 22nd, 2020 U. S. Food and Drug Administration approved the first drug remdesivir for use in adults and pediatric patients for the treatment of COVID-19 requiring hospitalization (11). This was followed by the development of other antiviral and anti-inflammatory drugs (12) and, finally, approving COVID-19 vaccines (13). On the other hand, patients hospitalized with COVID-19 often also receive antimicrobial treatment (7, 14, 15), which, of course, increases the threat of global antimicrobial resistance (3).

The aim of our study was to examine the consumption of antibiotics and the occurrence of antimicrobial resistance (AMR) infection and colonization in the selected general hospital in the observed period before and during the COVID-19 pandemic. We aimed to determine whether there is a causal relationship between the use of antibiotics and the increase in health care-associated infections (HCAI) and colonization with AMR, related to the COVID-19 pandemic.

## Materials and methods

### Study design and settings

The study was conducted in a secondary care regional hospital with 205 beds in Slovenia, which provides comprehensive hospital and specialist outpatient care at the secondary level for 110,000 inhabitants of Spodnje Podravje. During the COVID pandemic, the hospital had a COVID ward with 40 beds and an intensive care unit for the treatment of COVID-19 patients (5 beds). In 2021, the hospital also remained a COVID-19 hospital with part of its capacity dedicated to patients with COVID-19. 760 patients with COVID were treated in 2021.

### Data

We monitored the data in the period before the COVID-19 pandemic (2018, 2019) and during the COVID-19 pandemic (2020, 2021). Data on antibiotics consumption were obtained through the hospital pharmacy divided into basic group of antibiotics (Penicilin group: penicillin G, oxacillin, ampicillin, amoxicillin with clavulanic acid; Cephalosporine group: ceftriaxone, cefuroxime, cephalothin, cefixime; Sulphonamides group: sulfamethoxazole/trimethoprim; Aminoglycoside group: gentamicin; Macrolide group: erythromycin, azithromycin; Lincosamide group: clindamycin; Tetracycline group: tetracycline; Nitroimidazole group: metronidazole and mupirocin) and the second “reserve group” of antibiotics (Penicillin group: piperacillin/tazobactam; Cephalosporine group: ceftazidime/avibactam; ceftazidime, cefepime, cefotaxime; Carbapenem group: imipenem/cilastatin, meropenem, ertapenem; Aminoglycoside group: amikacin; Fluoroquinolone group: ciprofloxacin, levofloxacin, moxifloxacin; Glycopeptide group: vancomycin, teicoplanin; Oxazolidinone group: linezolid; Polymyxin group: colistin). The exact division and representatives of the basic and reserve groups of antibiotics are shown in Table 1.

**Table 1:** Division and representatives of the basic and reserve groups of antibiotics.

Antibiotic group	Basic group/representatives	Reserve group/representatives
Penicillin	Penicillin G, oxacillin, ampicillin, amoxicillin, amoxicillin with clavulanic acid	Piperacillin/tazobactam
Cephalosporine	Cefazolin, cefadroxil, cefalexin, cefaclor, cefprozil, cefuroxime, ceftriaxone	Cefotaxime, ceftazidime, ceftazidime/avibactam, cefixime, ceftibuten, cefepime
Aminoglycoside	Gentamicin	Amikacin
Macrolide	Erythromycin, azithromycin	
Lincosamide	Clindamycin	
Nitroimidazole	Metronidazole	
Sulphonamides	Sulfamethoxazole/trimethoprim	
Tetracycline	Tetracycline	
Carbapenem		Imipenem/cilastatin, ertapenem, meropenem
Glycopeptide		Vancomycin and teicoplanin
Oxazolidinone		Linezolid
Polymyxin		Colistin
Fluoroquinolone		Ciprofloxacin, moxifloxacin, levofloxacin

The unit of measure was defined as daily doses (DDD)/100 bed days. Data on AMR infections and colonization were obtained through the national laboratory for microbiological analysis of clinical samples on July 1st, 2023. No ethics committee approval or participant consent was received, as no human participants were involved. Fully anonymized aggregate data on the number and type of antibiotic-resistant bacterial infections and colonization in a selected hospital were obtained over a specific period. The data were accessed for research purposes, and the authors had no access to information that could identify individual patients during or after data collection.

### Statistical analysis

The data were processed in Microsoft Excel 2016 program and statistically analyzed in the IBM SPSS 20 program (IBM Corp. Released 2011. IBM SPSS Statistics for Windows, Version 20.0. Armonk, NY: IBM Corp.) using descriptive statistics, the Mann–Whitney U test, and Pearson or Spearman correlation test.

## Results

### Antibiotic consumption

In the two-year period before the COVID-19 pandemic (2018, 2019), 73.37% of the basic groups of antibiotics and 26.63% of the reserve groups of antibiotics were used. The changes during the pandemic (2020, 2021) were minimal, meaning 73.26% of the basic groups of antibiotics and 26.74% of the reserve groups of antibiotics were used.

**Figure 1:**
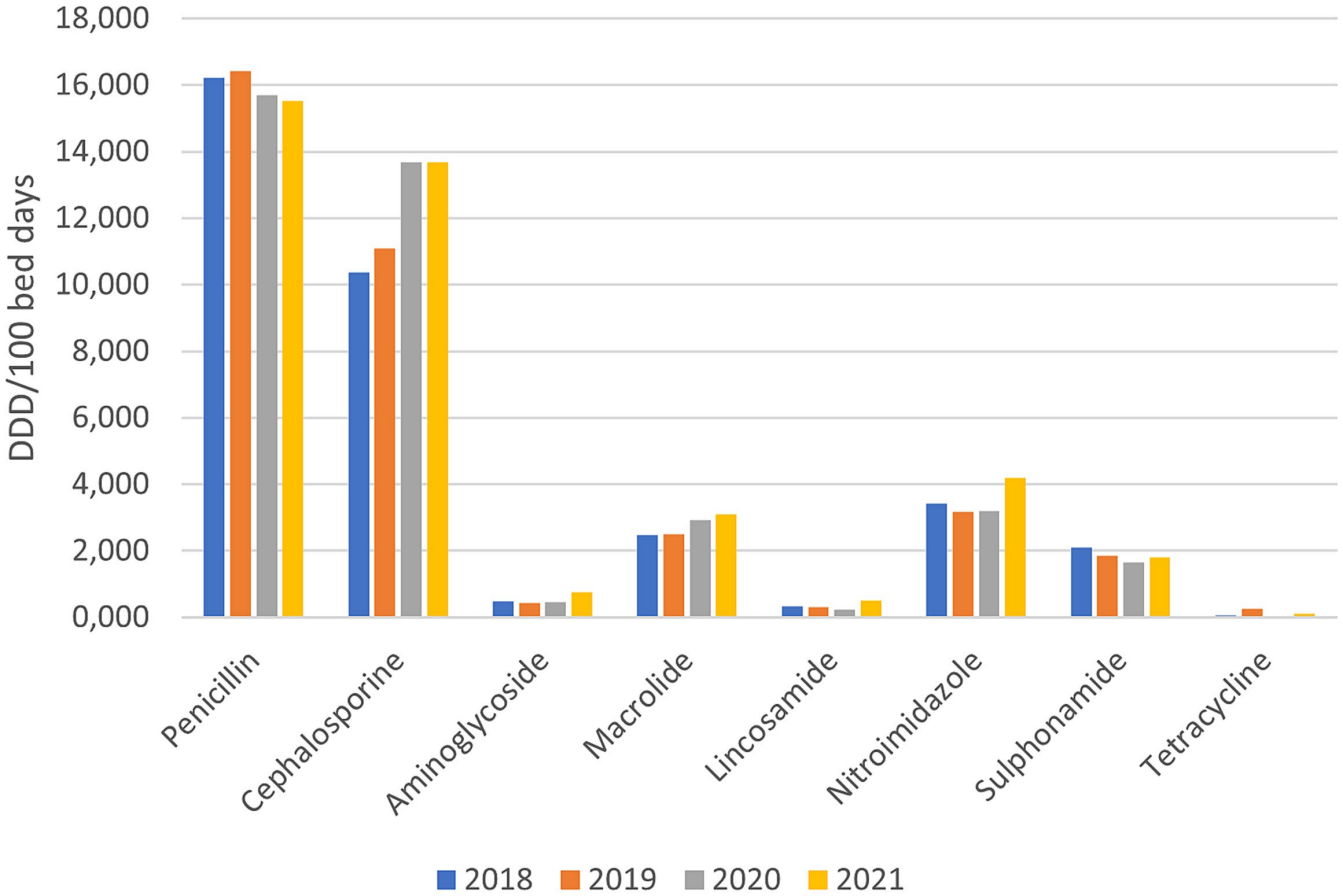
Consumption of basic groups of antibiotics before and during the pandemic (DDD/100 bed days).

The overall consumption of basic groups of antibiotics before and during the pandemic (Figure 1) shows the highest consumption of penicillin and cephalosporin antibiotics. The main difference between the two groups was revealed, i.e., the consumption of penicillin antibiotics decreased somewhat during the pandemic (−4.3%), while the consumption of cephalosporin antibiotics increased by +27.68%. In other basic groups of antibiotics increase during pandemic years was observed for aminoglycosides (+31.24%), macrolides (+21.08%), lincosamide (+14.86%) and nitroimidazole (+11.75%). In addition to penicillin, among the basic groups of antibiotics, consumption also decreased in the groups of sulphonamides (−12.79%) and tetracyclines (−63.27%).

**Figure 2:**
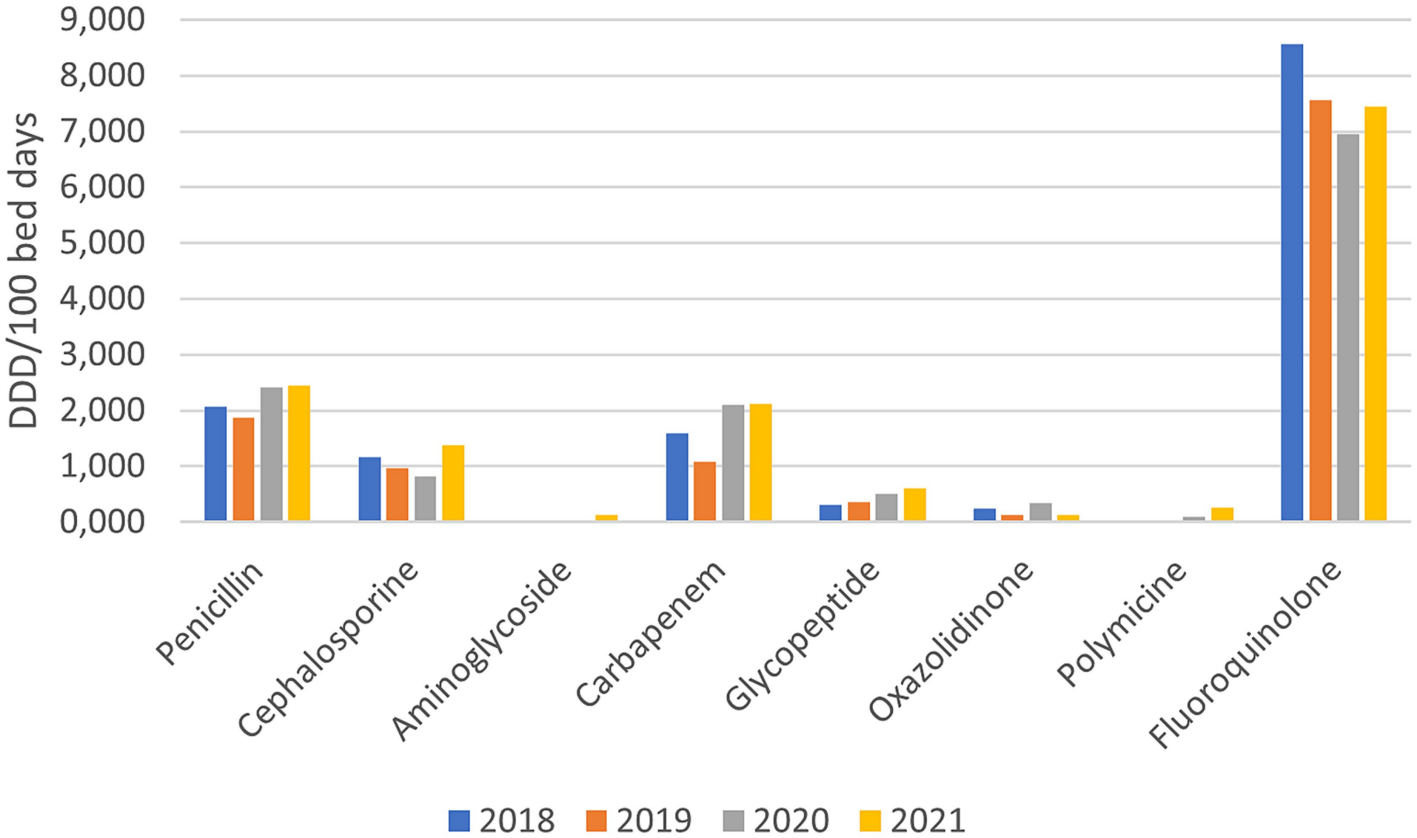
Consumption of reserve groups of antibiotics before and during the pandemic (DDD/100 bed days).

Among the reserve groups of antibiotics, fluoroquinolones are the most frequently prescribed, where a slight decrease (−10.79%) was observed during the pandemic years. In all the other reserve groups of antibiotics, an increased use was observed, namely penicillin’s - piperacillin/tazobactam (+23.42%), cephalosporins (+3.19%), carbapenems (+57.21%), Polymyxin (+1,030%), glycopeptides (+66.32%), oxazolidinone (+188.46%). The consumption of aminoglycosides (amikacin), which were not prescribed before the pandemic, grew to 0.129 DDD/100 bed days in 2021 (Figure 2).

**Figure 3:**
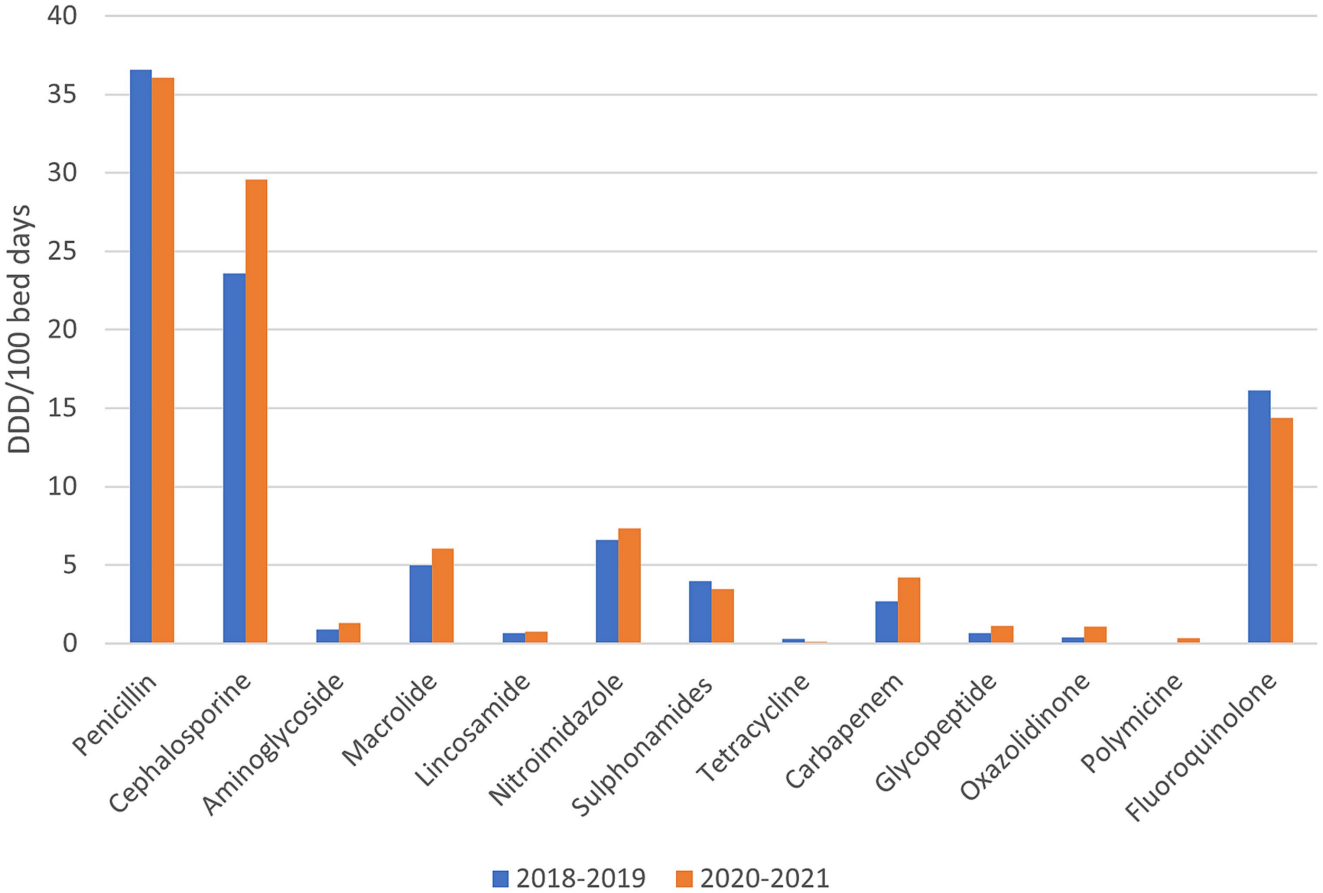
The consumption of all antibiotics (i.e., basic and reserve group) in the observed hospital before and during the pandemic.

In Figure 3, the consumption of all antibiotics (i.e., basic and reserve group) in the observed hospital before (years 2018, 2019) and during the pandemic (years 2020, 2021) is presented. During the COVID-19 pandemic, the consumption of penicillin antibiotics decreased by 1.39%, tetracyclines by 63.27%, sulphonamides by 12.79% and fluoroquinolones by 10.79%. On the other hand, the consumption of cephalosporins increased by 25.46%, aminoglycosides by 45.47%, macrolides by 21.08%, lacosamide by 14.86%, nitroimidazole by 11.75%, carbapenems by 57.21%, Polymyxin by 1,030% (10 x increase in consumption), glycopeptides by 66.32% and oxazolidinone by 188.46%.

The data analysis using the Mann–Whitney U Test did not show a statistically significant difference in total antibiotic consumption before and during the pandemic (*p* = 0.626).

### AMR infections and colonizations

The total occurrence of nosocomial infections and colonization with AMR bacteria in the observed hospital by individual years are shown in Table 2. All first cases (N) of newly discovered nosocomial infections and/or colonization in an individual patient, identified from clinical isolates and/or surveillance samples, are covered.

**Table 2:** Total nosocomial infections and colonization with AMR bacteria in the observed hospital by individual years.

	Before pandemic		During pandemic	
	2018	2019	2020	2021
MRSA	94	74	39	59
ESBL	192	169	187	224
CRAb	27	6	14	54
CRPs-CP	38	20	14	22
CRE-CPE	10	8	10	17
VRE	4	8	2	23

MRSA (Methicillin-resistant *Staphylococcus aureus*); ESBL (Extended Spectrum Beta-Lactamase); CRAb (Carbapenem-resistant *Acinetobacter baumannii*); CRPs-CP (Carbapenem-resistant Pseudomonas aerugionosa producing carbapenemase); CRE-CPE (Carbapenem-resistant enterobacteria producing carbapenemase); VRE (Vancomycin resistant Enterococci).

During the pandemic, the occurrence of Methicillin-resistant *Staphylococcus aureus* (MRSA) decreased by 41.67% and Carbapenem-resistant Pseudomonas aerugionosa producing carbapenemase (CRPs-CP) by 37.93%, despite the high consumption of penicillin, cephalosporins and higher consumption of carbapenems. This resulted in an increased occurrence of ESBL by 13.85%, Carbapenem-resistant *Acinetobacter baumannii* (CRAb) by 106.06%, Carbapenem-resistant enterobacteria producing carbapenemase (CRE-CPE) by 50% and Vancomycin resistant Enterococci (VRE) by 108.33% compared to the period before the COVID-19 pandemic.

The data analysis using the Mann–Whitney U Test did not show a statistically significant difference in the occurrence of AMR infections and colonizations before and during the pandemic (*p* = 0.631).

Using a Spearman correlation test, the connection between total antibiotic consumption and the occurrence of AMR infections and colonization was confirmed (*p* = 0.028).

Next, a correlation analysis of the consumption of specific antibiotics versus the occurrence of specific nosocomial infections and colonization with AMR bacteria in the observed hospital by individual years was performed and is shown in Table 3.

**Table 3:** Correlation coefficients (*r*) of specific antibiotics consumed versus occurrence of specific nosocomial infections and colonization with AMR bacteria.

	MRSA	ESBL	CRAb	CRPs-CP	CRE-CPE	VRE
Penicillin	0.71	−0.8	−0.7	0.51	−0.85	−0.69
Cephalosporine	−0.85	0.66	0.52	−0.65	0.72	0.53
Aminoglycoside	−0.16	0.94	0.95	−0.01	0.99	0.94
Macrolide	−0.78	0.74	0.62	−0.57	0.79	0.59
Lincosamide	0.15	0.81	0.89	0.2	0.87	0.96
Nitroimidazole	0.12	−0.45	0.44	0.16	0.46	−0.27
Sulphonamide	−0.24	0.38	−0.12	−0.33	0.28	0.34
Tetracycline	0.33	-0.5	0.21	0.28	−0.31	0.29
Carbapenem	0.45	0.56	0.68	−0.14	0.49	0.56
Glycopeptide	−0.67	0.67	0.29	0.19	0.52	0.32
Oxazolidinone	0.58	−0.44	0.45	−0.28	0.27	0.22
Polymicine	−0.14	0.39	0.33	0.41	0.54	−0.21
Fluoroquinolone	0.11	0.23	−0.18	−0.29	−0.12	0.17

Yellow/orange/red hues indicate increasing positive correlation, light/dark green hues indicate increasing negative correlation.

Aminoglycoside and lincosamide consumption correlated strongly (0.94 ≤ *r* ≤ 0.99) with multiple resistance types, including Extended Spectrum Beta-Lactamase (ESBL), CRAb, CRE-CPE, and VRE, suggesting a higher risk of resistance associated with their use. Carbapenem and glycopeptide consumption showed moderate positive correlations (0.52 ≤ *r* ≤ 0.68) with resistance, particularly with CRE-CPE, CRAb, and VRE. Penicillin and fluoroquinolone consumption generally displayed weaker or negative correlations (−0.80 ≤ *r* ≤ −0.12) with resistance types like ESBL and CRE-CPE, suggesting a lower association with resistance. The data showed mixed correlations with occurrences of MRSA, with some antibiotic use (e.g., penicillin and macrolide) correlating negatively, while other usage (e.g., carbapenem, oxazolidinone) showed weak positive correlations.

## Discussion

Misuse and overuse of antibiotics led to complex global public health challenges (16) way before the COVID-19 pandemic appeared. In the late 1990s and 2000, the WHO organized various meetings to address the public health threat of antimicrobial resistance, leading to the publication of the Global Strategy for Containment of Antimicrobial Resistance in 2001 (17). With the emergence of the COVID-19 pandemic, antibiotic misuse and AMR renewed their attention (6). It is estimated that 75% of patients with COVID-19 worldwide receive antibiotic prescriptions, although less than 10% of hospitalized and community-based patients are diagnosed with secondary bacterial infection requiring antibiotics (2, 14, 18–21).

Analyzing the data on antibiotic consumption in a selected Slovenian general hospital, the overall prescription of antibiotics stayed approximately the same, although some other studies report of increase in overall hospital antibiotic consumption in 2020 compared with that in 2019 (8, 22–26). However, some important differences can be observed when analyzing specific groups of antibiotics.

### Penicillin

Among penicillin antibiotics, basic group representatives’ consumption decreased, while reserve penicillin antibiotics (i.e., piperacillin/tazobactam) increased by as much as 23.42%. This somehow correlates with other research (8, 27) reporting piperacillin/tazobactam as one of the most frequently prescribed antibiotics during the COVID-19 pandemic, being a broad-spectrum antibiotic promising in treating very sick intensive care unit (ICU) admitted patients often requiring ventilatory support (28).

### Cephalosporin

Another group of antibiotics with observed increased consumption were cephalosporins (+25.46%) being repurposed during the pandemic (8, 19, 29). Despite increased consumption of penicillin and cephalosporin antibiotics, the occurrence of nosocomial infections and colonization with MRSA decreased by 41.67%. However, a statistically significant decrease in MRSA cases in EU countries was already observed between 2015 and 2019 (30), which is the consequence of improved infection prevention strategies and control (31) and prudent antimicrobial use (32).

### Macrolide

In our study, the consumption of macrolides increased by 21.08%. Nandi et al. (6) reported that the sales of macrolide antibiotics increased by 1.5% when the COVID-19 cases increased by 10% in the month since azithromycin was the most common antimicrobial agent used while treating COVID-19 (33). Although, at the beginning of the pandemic, azithromycin was believed to have some action against the SARS-COV-2 virus (34), subsequent research disproves this assumption (35). Cephalosporins and macrolides are being reported as the most common classes of antibiotics used during COVID-19 pandemic (28, 36–39), known to be effective against opportunistic pathogens that could cause secondary infection in COVID-19 (28).

### Carbapenems, polymyxin, glycopeptides, oxazolidinone

Regarding other reserve groups of antibiotics (i.e., carbapenems, polymyxin, glycopeptides, oxazolidinone), increased consumptions were observed, the most significant in 10 x increase consumption for polymyxin antibiotics, similar to some other studies (8, 40, 41). The increased use of reserve groups of antibiotics raises concerns (41) since those are meant to be used for treating infections with high-priority pathogens on the WHO list (42) or infections in the ICU (43, 44). Another concern associated with the increased use of carbapenem antibiotics is the rise of carbapenem-resistant and/or carbapenemase-producing isolates (i.e., ESBL, CRAb, CRPs-CP, CRE-CPE), being observed in our study and also reported by others (45, 46). The most significant increase was observed for CRAb, also demonstrated in other studies (47–53), which may be due to higher use of immunosuppressive therapy, invasive devices like ventilators and catheters, and more ICU admissions (54, 55). A similar observation was found for VRE infections and colonization being correlated to the increased use of vancomycin, but this could also be a continuation of the rising trend of VRE infections before the pandemic (14, 30, 56).

### Correlation analysis of antibiotic use and resistance

Our analysis found strong correlations between several antibiotics and resistance types. Aminoglycosides demonstrated high positive correlations with ESBL, CRAb, CRE-CPE, and VRE, indicating that increased aminoglycoside consumption may contribute to resistance in these organisms (57, 58). Similarly, lincosamides strongly correlated with VRE, suggesting a link between lincosamide use and resistance development (59). Carbapenems exhibited moderate to strong correlations with CRAb, CRE-CPE, and VRE, consistent with previous research associating carbapenem use with carbapenem-resistant bacteria (60, 61). Glycopeptides showed moderate positive correlations with ESBL and CRE-CPE, though to a lesser extent than aminoglycosides.

In contrast, penicillins and fluoroquinolones displayed weak to negative correlations with resistance, particularly with ESBL and CRE-CPE (62–64). This may suggest that these antibiotics pose a lower resistance risk in this context, though caution remains warranted given their potential resistance effects in other settings (35). The research’s limitations are that the data were collected in only one hospital, so we cannot generalize them more widely. Also, the data on prescribed antibiotics was collected, which does not necessarily reflect actual consumption. We should take into account that in 2020/2021, there were several waves of COVID-19, and the hospital situation changed every month, so perhaps certain effects of the epidemic are lost in the review of results on an annual basis.

## Conclusion

Although there are differences between antibiotic use and the occurrence of AMR bacteria both before and during the COVID-19 pandemic, the Centers for Disease Control and Prevention (CDC) reported that the pandemic caused a loss of progress in combating AMR (65) and the latest WHO evidence shows that the extensive overuse of antibiotics during COVID-19 pandemic worldwide may have worsened the spread of AMR (66). Such observations were also found in the Romanian hospital, where, despite a lower number of patients, an increased use of antibiotics was observed in 2021 (67). Even though we did not detect an increase in the overall consumption of antibiotics, there remains a worrying increase in certain important groups of antibiotics (the so-called representatives of the last line of defense: vancomycin, linezolid, piperacillin/tazobactam, meropenem, colistin). Similar was observed in the Romanian hospital where they also noticed a decrease in the proportion of antibiotics from the Watch and Reserve class and an increase in the proportion of antibiotics from the Access class (67). In our research, we did not observe the difference in the occurrence of AMR infections and colonizations before and during the pandemic. But we did observe an alarming increase in CRaB, ESBL and VRE and, in particular, highlight the increase in all AMR groups between the first and second year of the pandemic (2020, 2021). If in the first year of the pandemic (2020), the impact of the long-term effort to limit AMR may still be present, in the second year of the pandemic (2021), an extraordinary spread of AMR is already visible, which may also be the result of pandemic fatigue among employees. Nevertheless, the connection between antibiotic consumption and the occurrence of AMR infections and colonization was confirmed (*p* = 0.028).

## Data Availability

The original contributions presented in the study are included in the article/supplementary material, further inquiries can be directed to the corresponding author.
